# To operate or not to operate on women with deep infiltrating endometriosis (DIE) before *in vitro* fertilization (IVF)

**DOI:** 10.5935/1518-0557.20170027

**Published:** 2017

**Authors:** Márcia Mendonça Carneiro, Luciana Maria Pyramo Costa, Ivete de Ávila

**Affiliations:** 1 Endometriosis Multidisciplinary Team, Mater Dei Hospital, Belo Horizonte, MG, Brazil; 2 Human Reproduction Center at Mater Dei Hospital, Belo Horizonte, MG, Brazil; 3 Department of Obstetrics and Gynecology, Federal University of Minas Gerais, Brazil; 4 Endometriosis Multidisciplinary Team, Biocori Hospital, Belo Horizonte, MG, Brazil

**Keywords:** deep infiltrating endometriosis, *in vitro* fertilization, surgical treatment, infertility

## Abstract

Deep infiltrating endometriosis (DIE) can cause infertility and pelvic pain. There is little evidence of a clear connection between DIE and infertility, and the absolute benefits of surgery for DIE have not been established. This paper aimed to review the current literature on the effect of surgery for DIE on fertility, pregnancy, and IVF outcomes. Clinicians should bear in mind that a comprehensive clinical history is useful to identify patients at risk for endometriosis, although many women remain asymptomatic. Imaging can be useful to plan surgery. The effect of surgery on the fertility of women with DIE remains unanswered due to the heterogeneous nature of the disease and the lack of trials with enough statistical power and adequate follow-up. Surgery is not recommended when the main goal is to treat infertility or to improve IVF results. Decisions should be tailored according to the individual needs of each woman. Patients must be provided information on the potential benefits, harm, and costs of each treatment alternative, while the medical team observes factors such as presence of pelvic pain, patient age, lesion location, and previous treatments. In this scenario, management by a multidisciplinary endometriosis team is a key step to achieving successful outcomes.

## INTRODUCTION

Endometriosis is a benign gynecological disorder characterized by the presence and growth of endometriumlike tissue in sites outside the uterine cavity, primarily on the pelvic peritoneum and ovaries (Giudice & Kao, 2004; Bulun, 2009). It affects 5-10% of women of reproductive age and the main clinical features are chronic pelvic pain and infertility (Gupta *et al.*, 2008; de Ziegler *et al.*, 2010; Carneiro *et al.*, 2010).

Endometriosis seemingly causes infertility by interfering with fundamental steps in the reproductive process. The mechanisms of infertility associated with endometriosis remain controversial. Current evidence suggests that endometriosis adversely affects ovarian and tubal function as well as uterine receptivity resulting in female infertility. Endometriosis may also distort pelvic anatomy and produce large ovarian masses (Gupta *et al.*, 2008; de Ziegler *et al.*, 2010; Burney & Giudice, 2012). It is not clear, however, whether the products of endometriosis are a cause of infertility or a para-phenomenon.

Apparently, there are three types of endometriosis: superficial endometriosis, ovarian endometrioma, and deeply infiltrating endometriosis (DIE) (Tosti *et al.*, 2015). DIE is considered a specific entity which has been arbitrarily defined in histological terms as endometriotic lesions extending more than 5mm underneath the peritoneum (Cornillie *et al.*, 1990; Koninckx *et al.*, 1991). DIE is responsible for painful symptoms, whose severity has been strongly correlated with the anatomical location of the DIE lesions (Ávila *et al.*, 2016).

Present treatment options of endometriosis-associated infertility include surgery, superovulation with intrauterine insemination, and *in vitro* fertilization (IVF) (de Ziegler *et al.*, 2010; American Society for Reproductive Medicine (ASRM), 2012; Johnson & Hummelshoj, 2013). Although many questions remain unanswered, there is evidence to support the use of laparoscopic surgery to improve fertility. (Vercellini *et al.*, 2009; Berlanda *et al.*, 2013).

In addition, some studies suggest that the presence of endometriosis per se may adversely affect pregnancy and neonatal outcomes (Brosens *et al.*, 2012; Vigano *et al.*, 2015; Jacques *et al.*, 2016), while a recent systematic review stated that complications of endometriosis during pregnancy were rare and there was no evidence that the disease had a major detrimental effect on pregnancy outcomes (Leone Roberti Maggiore *et al.*, 2016). Women with incomplete surgical removal of posterior DIE may nevertheless present with a higher of risk complications during pregnancy and delivery. (Exacoustos *et al.*, 2016).

The role of surgical treatment in infertile women with endometriosis remains elusive (Vercellini *et al.*, 2009; Douay-Hauser *et al.*, 2011; Berlanda *et al.*, 2013). With the exception of peritoneal disease, randomized trials have not looked into the effects of surgery in subfertile women with endometriosis (Dufy *et al.*, 2014). Therefore, it has not been possible to define the absolute benefit of surgery for ovarian and rectovaginal lesions (Vercellini *et al.*, 2012; Berlanda *et al.*, 2013). The dilemma remains when we are faced with an infertile woman suffering from DIE: should they be offered first-line IVF treatment or surgery?

## METHODS

This paper aimed to review the current literature on the effect of surgery for DIE on fertility and IVF outcomes. A search was carried out on the Cochrane Library (July 2016) and PUBMED (1966 to July 2016) for relevant papers written in English, Italian, Spanish or French, which were the languages the authors could read. Combinations of medical subject heading terms including "deep infiltrating endometriosis and assisted reproductive technologies," "deep infiltrating endometriosis and infertility," "*in vitro* fertilization," "pregnancy," "intestinal endometriosis," and "urinary endometriosis" were used. The authors also looked for systematic reviews and Guidelines from the European Society of Human Reproduction and Embryology (ESHRE) and the American Society for Reproductive Medicine (ASRM), and books. The purpose was to find evidence to answer the following clinical questions: how should DIE be diagnosed in infertile women? How might DIE affect fertility and pregnancy? What are the possible benefits of surgery for DIE before IVF?

## RESULTS AND DISCUSSION

The search yielded 1,206 papers. The authors read the abstracts and excluded irrelevant papers and repeated titles appearing in more than one of the search combinations used. The full-text papers in the resulting list were obtained and read. The final list of relevant publications contained 60 papers.

### How should DIE be diagnosed in infertile women?

Comprehensive clinical history is useful to identify patients at risk for endometriosis, although establishing the diagnosis of the disease based solely on risk factors may be misleading, as a large portion of women with endometriosis remain completely asymptomatic (Ballard *et al.*, 2010; Carneiro *et al.*, 2013).

Finding women at risk may help identify the individuals who might benefit from diagnostic laparoscopy. Clinical history and symptom reports alone cannot be relied on to screen women with chronic pelvic pain. Preoperative questionnaires exploring four clinical symptoms of women with DIE associated with endometriomas may help identify high-risk groups (Lafay Pillet *et al.*, 2014). Individuals categorized in these groups should be referred to specialized centers for thorough assessment (Carneiro *et al.*, 2013; Dunselman *et al.*, 2014). Early diagnosis of DIE reduces care costs and allows the use of effective medical and surgical treatments to manage symptoms and improve the long-term outcome for patients. DIE should be considered as a diagnostic possibility in women with painful nodules/pain in areas of the rectovaginal wall or with visible nodules in the posterior vaginal fornix (Dunselman *et al.*, 2014).

Ultrasound has only recently been introduced in the diagnosis of DIE. Several studies provide enough evidence to show that transvaginal ultrasound (TVUS) is not only useful, but a strategic tool in the preoperative mapping of lesions and in surgery planning (Ferreira & Carneiro, 2010; Dunselman *et al.*, 2014; Exacoustos *et al.*, 2016).

As it is a noninvasive, readily available test in most centers, TVUS plays a pivotal role in the assessment of women with endometriosis along with bimanual pelvic examination. (Abrão *et al.*, 2007; Hudelist *et al.*, 2011;). Studies revealed that TVUS, when performed by skilled professionals, clearly enhances diagnostic accuracy, especially in patients with ovarian endometriomas or DIE involving the uterosacral ligaments, bladder or rectosigmoid junction; TVUS appears to be equally efficient in cases of DIE affecting the vagina and the pouch of Douglas (Hudelist *et al.*, 2011; Guerriero *et al.*, 2015; Reid & Condous, 2017).

Nisenblat *et al.* (2016) published a Cochrane Review in an attempt to establish the role of imaging in the diagnosis of endometriosis. The authors looked into 49 studies: ten involving endometriomas, 15 on DIE, and 33 looking at endometriosis at specific anatomical sites. Unfortunately, the majority of the studies were of poor methodological quality and none of the imaging methods studied was able to replace surgery in the diagnosis of endometriosis. The ESHRE 2014 Endometriosis Guidelines states that imaging may help define the extent of involvement in women with DIE. In addition to detecting endometriomas, magnetic resonance imaging (MRI) is an option when rectosigmoid endometriosis is considered.

Clinical practice still lacks accurate noninvasive diagnostic tests for endometriosis. A recent Cochrane review (Nisenblat *et al.*, 2016) evaluated possible noninvasive imaging tests and biomarkers for the diagnosis of endometriosis, but the authors were unable to find clinically useful biomarkers, and the evidence available was either insufficient or of poor-quality. Therefore, laparoscopy is still the gold standard for the diagnosis of endometriosis and the use of noninvasive tests either alone or in combination should only be considered in research settings.

Clinicians should therefore bear in mind that comprehensive clinical history is useful to find patients at risk for endometriosis, although establishing the diagnosis of the disease based solely on risk factors might be misleading, as a significant portion of women with endometriosis remain completely asymptomatic (Carneiro *et al.*, 2013).

Despite its low sensitivity and specificity, vaginal examination and evaluation of specific symptoms should not be completely ruled out in the basic diagnosis of endometriosis or in the planning of further therapeutic interventions (Carneiro *et al.*, 2013; Ávila *et al.*, 2016).

TVUS is a reproducible method used in the assessment of pelvic endometriosis severity that offers good agreement with laparoscopy findings. Other imaging techniques such as MRI are suitable for the diagnosis of endometriosis, but it is our belief that TVUS is the preferred initial imaging mode, since it is readily available in most centers and offers an easy access, affordable and accurate means of diagnosing patients with ovarian endometriomas and DIE (Benacerraf & Groszmann, 2012; Dunselman *et al.*, 2014).

The decision to perform surgery for deep endometriosis is mainly clinical. TVUS and other imaging techniques such as MRI may be useful in the preoperative estimation of lesion size and lateral extension, and play a vital role in surgical planning and choice of approach. It remains unclear, however, the extent to which preoperative ultrasonography or MRI should influence the decision to perform surgery, or the choice of procedure to treat deep endometriosis (Carneiro *et al.*, 2013).

### How might DIE affect fertility and pregnancy?

Although DIE has been frequently associated with infertility, there is little evidence connecting the disease and infertility. Studies suggest that infertility in women with DIE is probably related to the strong link between DIE and adhesions, superficial endometriotic implants, ovarian endometriomas, and adenomyosis (Somigliana & Garcia-Velasco, 2015).

Several anomalies are thought to contribute to DIE-related infertility: hormonal function (estrogen and progesterone receptors) and immunological factors, such as peritoneal macrophages, natural killer cells, and lymphocytes (Gupta *et al.*, 2008; de Ziegler *et al.*, 2010; Burney & Giudice, 2012).

Unfortunately, the relationship between DIE and infertility is rather complex and a final conclusion on the matter yet to be produced (Somigliana & Garcia-Velasco, 2015). When considering infertility in women with DIE, clinicians must bear in mind a variety of confounding factors that might interfere with the interpretation of studies. Firstly, other types of endometriosis, such as peritoneal and ovarian, may accompany DIE and affect fertility on their own (de Ziegler *et al.*, 2010). Isolated DIE is rarely encountered, whereas associations with other forms of endometriosis are frequently seen (Somigliana *et al.*, 2004). Secondly, superficial peritoneal lesions may produce inflammatory cytokines and chemokines, and thus produce, in an altered hormonal milieu, increased oxidative stress and impaired sperm and tubal function (Gupta *et al.*, 2008; de Ziegler *et al.*, 2010). In addition, endometriomas may interfere with folliculogenesis and result in poor oocyte and embryo quality and impair ovarian response and pregnancy rates in IVF (Ballester *et al.*, 2012; Yang *et al.*, 2015). Adenomyosis, a finding more frequent in women with DIE than other forms of endometriosis, might also significantly reduce (68%) the likelihood of pregnancy in women attempting conception after surgery for rectovaginal or colorectal endometriosis (Di Donato *et al.*, 2014; Vercellini *et al.*, 2014). And last but not least, published data suggests the endometrium itself harbors several anomalies in women with endometriosis, which result in reduced endometrial receptivity and decreased implantation rates (Gupta *et al.*, 2008; de Ziegler *et al.* , 2010).

### What is the impact of surgery on the fertility of women with DIE?

DIE is a rather heterogeneous disease. Analysis of the anatomical location of the lesions revealed a multifocal pattern, as 61% of the women with DIE had more than one site simultaneously affected (Ávila *et al.*, 2016). The multifocal distribution pattern observed in women with DIE makes it more difficult to study possible relationships between pain and anatomical location, or to establish its role on fertility (Ávila *et al.*, 2016). Besides, there is a paucity of data from randomized controlled trials, as the available published studies are case series or uncontrolled studies with many confounding factors such as use of ART and presence of other infertility factors.

Natural fecundity in women with endometriosis is rarely evaluated, and except for peritoneal disease, no randomized trials have been carried out to assess the effect of surgery in this setting (Somigliana & Garcia-Velasco, 2015).

Brown & Farquhar (2014) compiled evidence from 17 Cochrane Systematic Reviews on treatment options for women with pain or subfertility associated with endometriosis using live birth, clinical pregnancy, ongoing pregnancy, miscarriage and adverse events as primary outcomes. Seven reviews concerned infertility: two presented ART-related outcomes (Sallam *et al.*, 2006 and Benschop *et al.*, 2010), while the remaining described spontaneous pregnancy. Post-surgical medical treatment resulted in no benefits in terms of pregnancy rates, but three months of treatment with GnRH agonists improved pregnancy rates in women with endometriosis undergoing IVF. Excisional surgery resulted in better spontaneous pregnancy rates in the 9-12 month period after surgery when compared to ablative surgery. Laparoscopic surgery improved live birth and pregnancy rates when compared to diagnostic laparoscopy alone. Medical treatment apparently did not improve clinical pregnancy rates.

Duffy *et al.* (2014) found that laparoscopic surgery was associated with an increased live birth or ongoing pregnancy and clinical pregnancy rates in comparison to diagnostic laparoscopy. No solid conclusions of safety were drawn, as there was insufficient evidence on adverse events.

The two randomized controlled trials reported by Hart *et al.* (2008) suggested a benefit of excisional surgery over drainage or ablation of endometriomata to achieve pregnancy. Benschop *et al.* (2010) evaluated surgical interventions for endometrioma before IVF. The authors did not find evidence of a difference in clinical pregnancy rates between surgery (aspiration or cystectomy) for endometrioma prior to ART and expectant management.

While some advocate complete surgical removal of endometriotic lesions to improve fertility (Daraï *et al.*, 2005; Ferrero *et al.*, 2009), others affirm that extensive surgery for peritoneal endometriosis and DIE in infertile women does not improve global fertility prognosis and may be associated with higher complication rates (Vercellini *et al.*, 2006; Douay-Hauser *et al.*, 2011; Vercellini *et al.*, 2012). Vercellini *et al.* (2012) pinpointed in their literature review that women should be carefully counseled on the real chances of getting pregnant after surgery. The authors found that pregnancy rates decreased by 15% in individuals who sought spontaneous conception after surgery versus women offered IVF (39% vs. 24%). Apparently, time to conception after surgery is also a matter to consider, as delays have been associated with lower pregnancy and higher relapse rates (Somigliana *et al.*, 2010).

In short, the effect of surgery on the fertility of women with DIE remains unanswered due to the heterogeneous nature of the disease and the lack of adequate trials with enough power and follow-up to study the matter. Surgery cannot be recommended when the main goal is to treat infertility, as the evidence to support such approach is still scant. Decisions should be tailored according to the individual needs of each woman after they are provided with information on the potential benefits, harm, and costs of each treatment alternative (Vercellini, 2015).

### What are the possible benefits of surgery for DIE before IVF?

According to the guidelines of the European Society of Human Reproduction and Embryology (ESHRE) (Dunselman *et al.*, 2014), there is limited evidence for performing surgery with the sole objective of increasing live birth rates. IVF is recommended in cases of infertility associated with endometriosis, if pelvic anatomy is distorted and tubal function impaired, or in cases of male factor infertility and/or other treatments have failed. The main purpose of surgery for women suffering from endometriosis-related infertility ideally revolves around the restoration of normal pelvic anatomical relationships and preservation of the function of pelvic organs.

Bianchi *et al.* (2009) published the only prospective study available to date. The authors aimed at comparing IVF results in women with DIE-associated infertility submitted to extensive laparoscopic excision of endometriosis before IVF to subjects not operated on before IVF. This prospective cohort study included 179 women divided into two groups: IVF only (n = 105) and surgical resection of DIE lesions before IVF (n=64). The odds of achieving pregnancy were 2.45 times higher in the group submitted to surgical excision before IVF.

DIE has been blamed for lowering the pregnancy rates of IVF cycles. Ballester *et al.* (2012) carried out a retrospective study and looked at 103 women with endometriomas (n = 30) and with endometriomas associated with DIE (n = 73). When associated with endometrioma, DIE adversely affected cumulative pregnancy rates (82.5% vs. 69.4%). The authors recommended surgery when pregnancy was not achieved after three attempts at IVF. Centini *et al.* (2016) also reported that the surgical removal of multiple lesions increased pregnancy and live birth rates both spontaneously and after IVF. On the other hand, Capelle *et al.* (2015) considered that surgery for DIE before IVF did not result in improved pregnancy and birth rates. Interestingly, infertile women with DIE trying to have a second child and who had had surgery had high live birth rates (78%) and a spontaneous pregnancy rate of 54% (Boujenah *et al.*, 2016).

The impact of surgery on IVF results remains controversial. While women with advanced-stage endometriosis submitted to surgery before IVF did not respond as well to gonadotropins when compared to women with tubal-factor infertility, the implantation, pregnancy, and delivery rates were nonetheless similar (Matalliotakis *et al.*, 2007). Complete elimination of all visible endometriotic lesions in women with minimal and mild endometriosis undergoing IVF may result in shorter time to pregnancy and higher live birth rates (Opøien *et al.*, 2011). Other authors have suggested that IVF pregnancy rates of women with DIE may be significantly improved after extensive laparoscopic excision of DIE (Bianchi *et al.*, 2009). A combination of surgery with IVF has been suggested as a more effective approach in endometriosis-associated infertility (Coccia *et al.*, 2008; Barri *et al.*, 2010; Ballester *et al.*, 2012; Capelle *et al.*, 2015). With the exception of patients with endometrioma, infertile women with various stages of endometriosis have enjoyed the same success rates with IVF as patients with tubal factor infertility (Opøien *et al.*, 2011).

The effect of surgery in this setting remains controversial. The available published studies are observational and are not large enough to allow for any definitive conclusions. Except for peritoneal disease, no randomized trials have been published to determine the effect of surgery in subfertile women with endometriosis (Brown & Farquhar, 2014). It remains therefore impossible to define the absolute benefit of surgery for ovarian and rectovaginal lesions. The decision to undergo surgery for endometriosis-associated subfertility must be thoroughly debated with the patient. Detailed information weighing risks and benefits, and other variables such as presence of pain, large or complex adnexal masses, bowel or ureteral stenosis, and coexisting infertility factors, must be discussed. Specifically in cases of recurrent endometriosis, IVF should generally be the first option (Vercellini *et al.*, 2012; ASRM, 2012; Berlanda *et al.*, 2013).

## CONCLUSION

The role of surgery in the treatment of infertile women with DIE remains a matter of intense debate. Available evidence is poor, as it amounts mostly from case series, which may bias possible conclusions. Therefore, a clear-cut conclusion cannot be drawn. Women with DIE should be counseled individually taking into consideration several factors such as presence of pelvic pain and other symptoms, age, lesion location, previous treatments (surgery and ART), as well as possible pregnancy complications. In this scenario, management by a multidisciplinary endometriosis team is a key factor in achieving successful outcomes.
